# Aflatoxin B1 exposure disrupts organelle distribution in mouse oocytes

**DOI:** 10.7717/peerj.13497

**Published:** 2022-05-23

**Authors:** Yan-Zhe Zhang, Qian-Han Zhao, Hong-Wei Duan, Yuan-Jing Zou, Shao-Chen Sun, Lin-Lin Hu

**Affiliations:** 1 College of Animal Science & Technology, Nanjing Agricultural University, Nanjing, China; 2 The Affiliated Hospital of Youjiang Medical University for Nationalities, Baise, China

**Keywords:** Aflatoxin B1, Oocyte, Organelles, Mitochondria, Meiosis

## Abstract

Aflatoxin B1 (AFB1) is a secondary metabolite produced by the fungus Aspergillus, which is ubiquitous in moldy grain products. Aflatoxin B1 has been reported to possess hepatotoxicity, renal toxicity, and reproductive toxicity. Previous studies have shown that AFB1 is toxic to mammalian oocytes. However, the potential toxicity of AFB1 on the organelles of mouse oocytes is unknown. In this study, we found that exposure to AFB1 significantly reduced mouse oocyte development capacity. Further analysis showed that the endoplasmic reticulum (ER) failed to accumulate around the spindle, and scattered in the cytoplasm under AFB1 exposure. Similar to the ER, the Golgi apparatus showed a uniform localization pattern following AFB1 treatment. In addition, we found that AFB1 exposure caused the condensation of lysosomes in the cytoplasm, presenting as a clustered or spindle peripheral-localization pattern, which indicated that protein modification, transport, and degradation were affected. Mitochondrial distribution was also altered by AFB1 treatment. In summary, our study showed that AFB1 exposure had toxic effects on the distribution of mouse oocyte organelles, which further led to a decline in oocyte quality.

## Introduction

Mycotoxins are naturally-occurring low molecular weight toxins produced by filamentous fungi as secondary metabolites. Mycotoxin disease is considered to be a typical example of “natural poisoning” (Bennett & Klich, 2003). Mycotoxins are commonly found in contaminated food, such as grains and fruits, and can cause illness and death in humans and other animals (Dai et al., 2019; Drusch & Ragab, 2003). The most common known mycotoxins include aflatoxins, fumonisins, ochratoxin, and aflavenone (Gallo et al., 2015). Aflatoxins are produced by fungi of the genus Aspergillus, especially *A. flavus*, which is widely distributed in soil and known to contaminate crops such as rice and corn (Rushing & Selim, 2019). Among them, Aflatoxin B1 (AFB1) is the strongest carcinogen naturally produced by Aspergillus, which is classified as a Group 1 human carcinogen by the International Agency for Research on Cancer (Sherif, Salama & Abdel-Wahhab, 2009). Previous studies have confirmed that AFB1 has serious deleterious effects on liver function (Chen, Ishfaq & Wang, 2021), kidney function (Li et al., 2018), and the reproductive system (Fouad et al., 2019). Aflatoxin B1 exposure can induce oxidative stress, apoptosis, and an inflammatory response, ultimately leading to liver injury (Limaye et al., 2018). Moreover, AFB1 (0.5 mg/kg) activates oxidative stress-related pathways and causes kidney damage (Li et al., 2018). In reproduction, AFB1 may completely or partially (dose-dependent) inhibit spermatogenesis, leading to sperm abnormalities and testicular atrophy (Ortatatli et al., 2002). And AFB1 can also reduce the maturation ability of oocytes in porcine, as well as cause apoptosis and oxidative stress (Liu et al., 2015). In mouse oocytes, AFB1 treatment has been shown to reduce the developmental potential oocytes to become embryos, and cleaved embryos to become blastocysts, resulting in lower embryo quality (Jiang et al., 2019). However, the toxic effects of AFB1 on organelle distribution and function of mammalian oocytes have not been reported.

In mammals, successful fertilization is based on maternal maturity and the quality of the oocyte. To ensure high levels of oocyte growth potential, correct spatial and temporal dynamics of organelles in oocytes; such as endoplasmic reticulum (ER), Golgi apparatus, lysosomes, and mitochondria are required (Sirard et al., 2006). The endoplasmic reticulum is a multifunctional organelle found in eukaryotic cells that regulates intracellular calcium and serves as a site for the synthesis of lipids and transmembrane proteins. The Golgi apparatus is an organelle that plays a central role in a variety of transport events related to protein delivery and modification (Mao et al., 2014). Lysosomes exist in the cytoplasm as membrane-bound organelles and participate in the autophagy pathway as autophagosomes (Luzio, Pryor & Bright, 2007). Mitochondria, primarily through oxidative phosphorylation, provide energy to cells in the form of adenosine triphosphate (ATP) (Martin, 2017). Previous studies have shown that ER stress has negative effects on oocyte maturation (Lin et al., 2019). In addition, a fully functional Golgi membrane is required for germinal vesicle rupture (GVBD), which is a morphological feature of meiosis recovery (Mao et al., 2014). Studies have also demonstrated that a gradual increase of lysosomal autophagy can significantly reduce the fertilization and developmental potential of mature oocytes *in vitro* (McGinnis, Pelech & Kinsey, 2014). In addition, stress-induced changes in mitochondrial function also lead to a reduction in oocyte maturation (Roth, 2018).

In the current study, we used a mouse model to explore the potential toxicity of AFB1 on the distribution of mammalian oocyte organelles including the ER, Golgi apparatus, lysosome, and mitochondria. Our results demonstrated that AFB1 was able to disrupt the cellular distribution of the ER, Golgi apparatus, lysosomes, and mitochondria, which may subsequently lead to the dysfunction of these organelles. Therefore, our study provided important evidence for the toxicity of AFB1 on organelle function in mammalian oocytes.

## Materials and Methods

### Ethics statement and oocyte culture

The Animal Research Committee of Nanjing Agricultural University in China authorized the study and our operations with mice followed the requirements (Su-XYXK-2017-011). Female 4-week-old ICR mice were kept at a constant temperature of 24 °C in a 12-h light-dark cycle and had unrestricted access to food and water during the research period. After the experiments, all mice were killed by cervical dislocation. We collected germinal vesicle intact oocytes from mouse ovaries in M2 medium (Sigma, St. Louis, MO, USA), washed them three times, and cultured the oocytes in M16 media (Sigma, St. Louis, MO, USA) for 8 h (metaphase I, MI) or 12 h (metaphase II, MII) under paraffin oil at 37 °C in a 5% CO_2_ environment.

### Aflatoxin B1 treatment

Aflatoxin B1 (CAS:1162-65-8, Macklin) was dissolved in DMSO to prepare a 40 mM stock solution and then diluted with M2 medium to the final concentrations of 100 and 200 μM, with the concentration of DMSO <1% in the culture medium. The oocytes were exposed to different concentrations of AFB1 at 37 °C, 5% CO_2_, and were cultured for 8 or 12 h. The same amount of DMSO was added to the control group.

### Mitochondria and ER detection

Mito-Tracker Red CMRos (1:600) (Cat# M7512; Invitrogen, Eugene, OR, USA) and ER-Tracker Red (1:500) (C1041; Beyotime Biotechnology, Shanghai, China) were used to identify the distribution of mitochondria and the ER in live oocytes in M2 medium at 37 °C for 30 min. After that, we washed the oocytes three times and immediately scanned the live oocytes with a confocal laser-scanning microscope (Zeiss LSM 800 META; Zeiss, Berlin, Germany).

### Golgi apparatus and lysosome detection

To detect Golgi apparatus, we first removed the zona pellucida by incubating the oocytes with 1% pronase for 4 min. The live oocytes were then incubated in M2 medium at 4 °C for 30 min with Golgi-Tracker Red (1:100) (C1043; Beyotime Biotechnology Shanghai, China). We incubated the oocytes with M2 at 37 °C for 30 min after washing them three times with fresh culture media. Finally, we used a confocal laser-scanning microscope (Zeiss LSM 800 META, Zeiss, Berlin, Germany) to analyze the oocytes.

Similarly, lysosome Red (1:10,000) (C1046; Beyotime Biotechnology, Shanghai, China) was used to identify lysosomal distribution in live oocytes treated with M2 medium at 37 °C for 30 min. The oocytes were cleaned three times before being examined by a confocal laser scanning microscope (Zeiss LSM 800 META; Zeiss, Berlin, Germany).

### Fluorescence intensity analysis

We analyzed the fluorescence intensity of the samples using Image J software (NIH). We fixed the control and treated oocytes in separate areas on the same live cell culture dish. In addition, we detected the mean fluorescence intensity per unit of area of the region of interest and then performed statistical analysis on the mean values of the control and treatment groups.

### Statistical analysis

GraphPad Prism 5 software (GraphPad, San Diego, CA, USA) was used to examine the data, and the independent sample t-test was employed for statistical analysis. Each experiment was carried out at least three times. Data were expressed as mean ± SEM. *P* < 0.05 was considered statistically significant.

## Results

### Effects of AFB1 on the developmental competence of mouse oocytes

**Figure 1:**
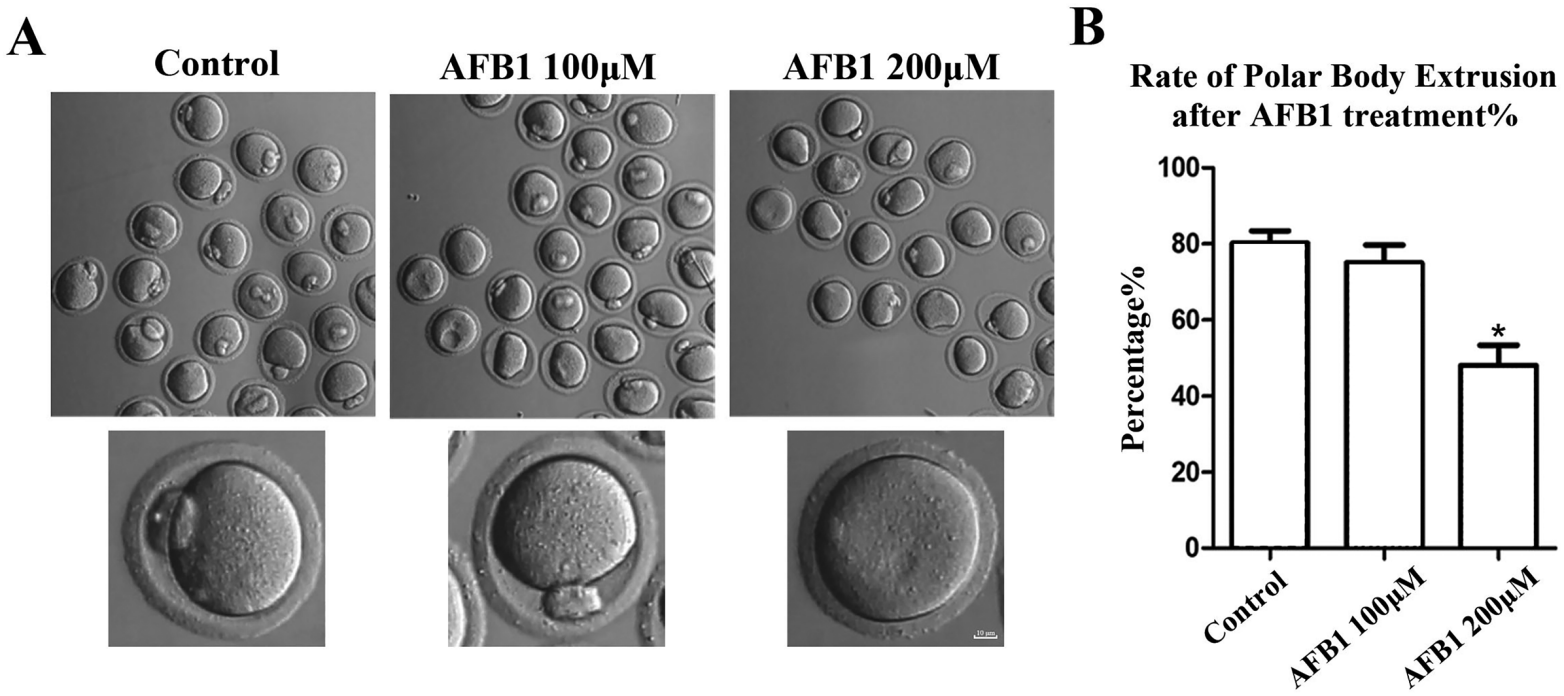
Effects of AFB1 on the developmental competence of mouse oocytes. (A) A typical picture of oocyte polar body extrusion following AFB1 exposure (control group, *n* = 94; 100 μM AFB1 group, *n* = 92; 200 μM AFB1 group, *n* = 83). Bar = 10 μm. (B) The rate of polar body extrusion following AFB1 exposure in mice. The rate of polar body extrusion was significantly lower than that of the control group after AFB1 exposure. *P = 0.04.

To investigate the effects of AFB1 on mouse oocytes, we first examined the maturation of oocytes exposed to AFB1 (concentrations of 100 and 200 μM) after 12 h in culture. As shown in Fig. 1A, in the control group, the majority of oocytes extruded the first polar body and reached the stage of metaphase II (MII) (80.17 ± 3.27%, *n* = 94). There was no significant difference for the 100 μM AFB1-exposed group compared with the control group (75.06 ± 4.66%, *n* = 92, *P* = 0.55). However, when compared to the control group, the rate of polar body extrusion was significantly lower in the 200 μM treatment group (48.01 ± 5.32%, *n* = 83, *P* = 0.04) (Fig. 1B). We thus selected the 200 μM AFB1 treatment condition for the subsequent study.

### Effects of AFB1 on ER distribution in mouse oocytes

**Figure 2:**
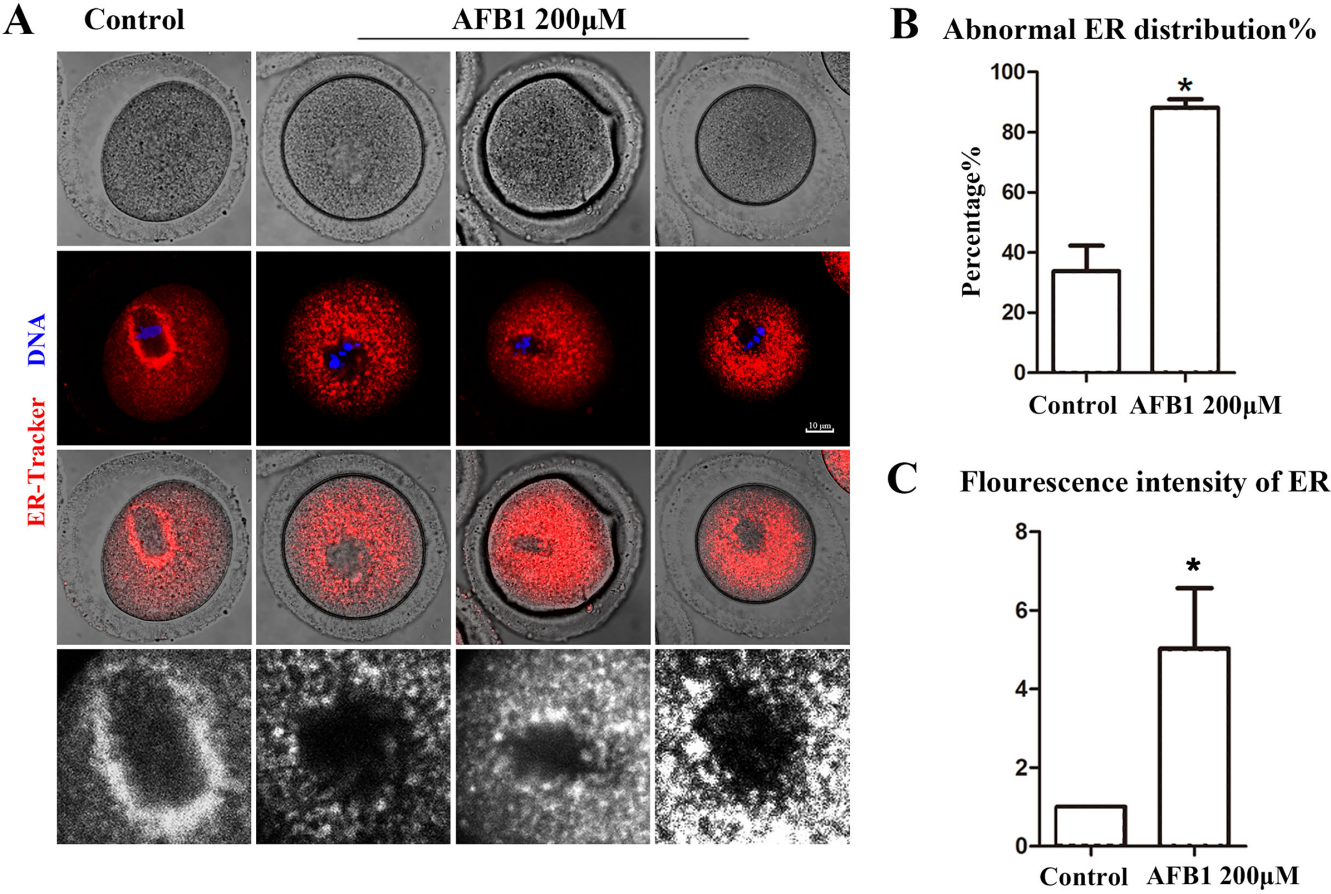
Effects of AFB1 on ER distribution in mouse oocytes. (A) A typical picture of the ER distribution in the control group and the AFB1-exposed group. Red, ER-tracker. Blue, DNA. Bar = 10 μm. (B) The rate of abnormal ER distribution was significantly increased in the 200 μM group (control group, *n* = 44; AFB1 group, *n* = 50). *P = 0.01. (C) The fluorescence intensity of the ER-Tracker dye was increased markedly in the 200 μM AFB1 group (control group, *n* = 31; AFB1 group, *n* = 34). *P = 0.12.

Protein biosynthesis takes place mostly in the ER. We utilized the ER-Tracker dye to examine the distribution of the ER following AFB1 exposure in mouse oocytes. In the control group, the ER in the MI stage was predominantly distributed around the spindle, while oocytes exposed to AFB1 mostly showed a homogenous distribution rather than a perinuclear distribution (Fig. 2A). The statistical analysis revealed that following AFB1 exposure, the aberrant rate of ER distribution rose dramatically (control group, 33.81 ± 8.47%, *n* = 44; AFB1 group, 88.15 ± 2.81%, *n* = 50, *P* = 0.01) (Fig. 2B). To further determine the effect of AFB1 on the ER, we calculated the fluorescence intensity of the ER-Tracker dye and found it to be markedly stronger in the treated group compared to control oocytes (control group, 1, *n* = 31; AFB1 group, 5.03 ± 1.54, *n* = 34, *P* = 0.12) (Fig. 2C). These results demonstrated that AFB1 exposure affected the distribution of the ER in mouse oocytes.

### Effects of AFB1 on Golgi apparatus distribution in mouse oocytes

**Figure 3:**
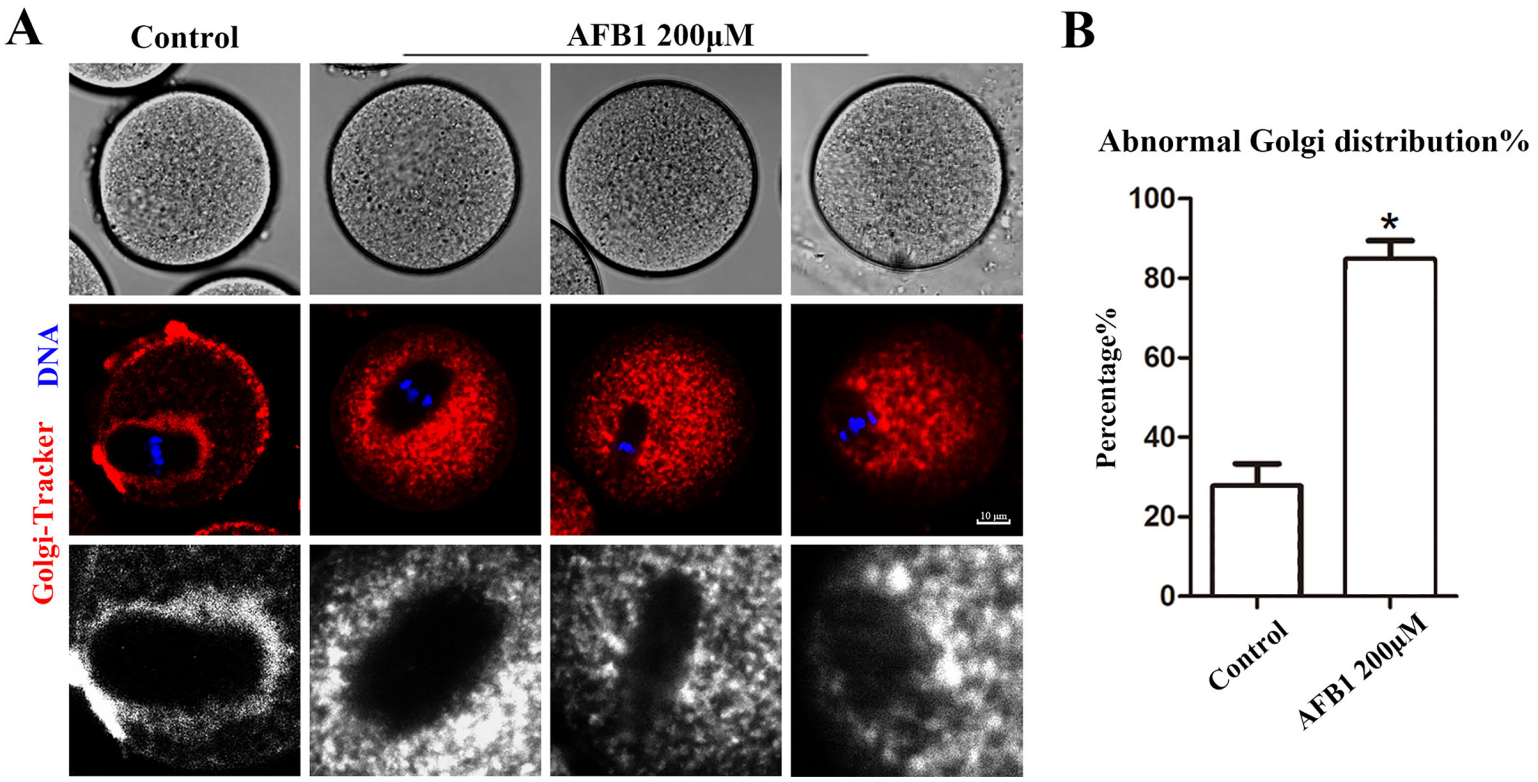
Effects of AFB1 on the Golgi apparatus distribution in mouse oocytes. (A) A typical picture of Golgi apparatus distribution in the control group and the AFB1-exposed group (control group, *n* = 45; AFB1 group, *n* = 44). Red, Golgi-tracker. Blue, DNA. Bar = 10 μm. (B) The rate of abnormal Golgi apparatus distribution was significantly increased in the 200 μM AFB1 group. *P = 0.03.

The Golgi apparatus is a docking site for cargo transit and is strongly tied to protein production and transport. Using the Golgi-Tracker dye, we examined the distribution of Golgi apparatus in mouse oocytes. As shown in Fig. 3A, similar to the ER, Golgi apparatus of control oocytes were also distributed around the spindle. In the treated group, Golgi apparatus failed to accumulate to the periphery of the spindle. In addition, compared with the control group, the abnormal distribution rate of Golgi apparatus was significantly increased after AFB1 treatment (control group, 27.74 ± 5.66%, *n* = 45; AFB1 group, 84.83 ± 4.59%, *n* = 44, *P* = 0.03) (Fig. 3B). These results suggested that AFB1 exposure disrupted the distribution of Golgi apparatus in mouse oocytes.

### Effects of AFB1 on lysosomal distribution in mouse oocytes

**Figure 4:**
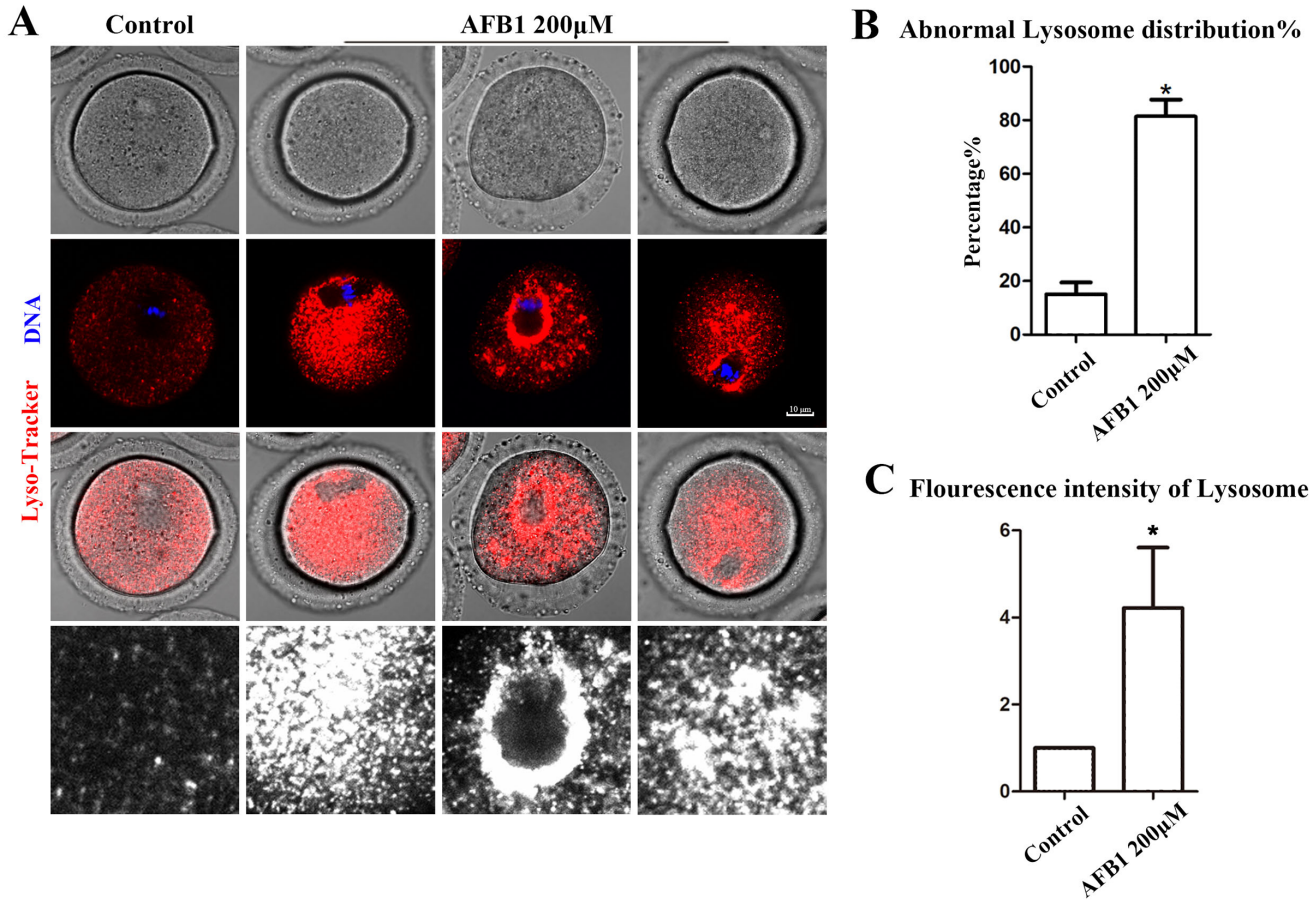
Effects of AFB1 on the lysosome distribution in mouse oocytes. (A) A typical picture of lysosomal distribution in the control group and the AFB1-exposed group. Red, lysosome. Blue, DNA. Bar = 10 μm. (B) The rate of abnormal lysosome distribution was significantly increased in the 200 μM AFB1 group (control group, *n* = 51; AFB1 group, *n* = 57). *P = 0.02. (C) The fluorescence intensity of lysosomes was increased markedly in the 200 μM AFB1 group (control group, *n* = 56; AFB1 group, *n* = 52). *P = 0.20.

Being organelles closely related to intracellular proteins, we also examined lysosomes to further support the disruptive effect of AFB1 on oocyte organelles. As shown in Fig. 4A, we found that the distribution of lysosomes in the control group was homogeneous in the cytoplasm, but in the treated group lysosomes were either agglutinated in the cytoplasm or aggregated around the spindle. In addition, compared with the control group, the rate of the abnormal distribution of lysosomes in the treatment group was significantly increased (control group, 15.05 ± 4.37%, *n* = 51; AFB1 group, 81.37 ± 6.37%, *n* = 57, *P* = 0.02) (Fig. 4B). Furthermore, we discovered that in the treatment group, the fluorescence intensity of lysosomes in the cytoplasm of oocytes was substantially higher than in the control group (control group, 1, *n* = 56; AFB1 group, 5.45 ± 2.36, *n* = 52, *P* = 0.20) (Fig. 4C). These results indicated that AFB1 exposure impaired the distribution of lysosomes in mouse oocytes.

### Effects of AFB1 on mitochondrial distribution in mouse oocytes

**Figure 5:**
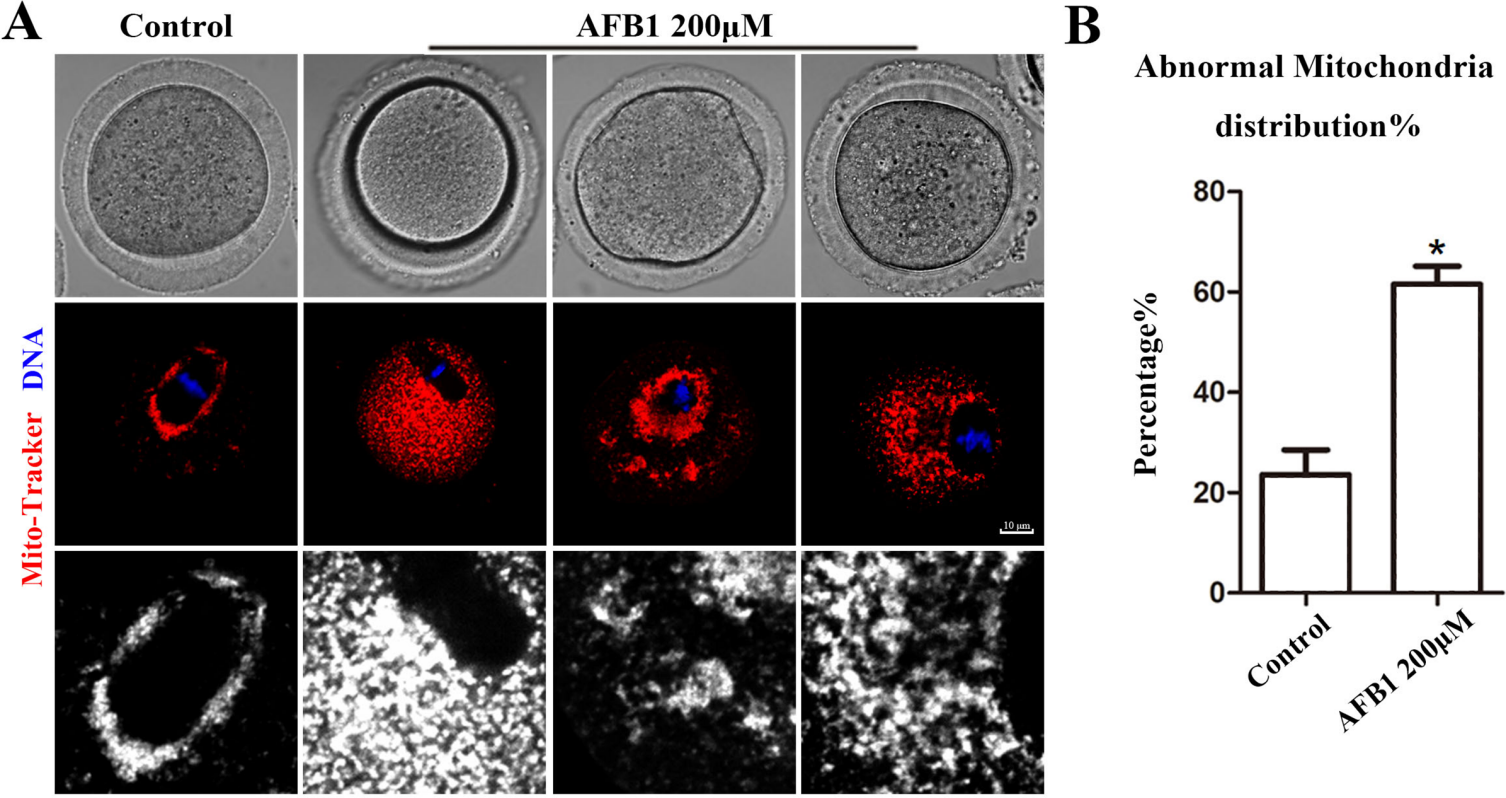
Effects of AFB1 on the mitochondria distribution in mouse oocytes. (A) A typical picture of the mitochondria distribution in the control group and the AFB1-exposed group (control group, *n* = 44; AFB1 group, *n* = 54). Red, mitochondria. Blue, DNA. Bar = 10 μm. (B) The rate of abnormal mitochondria distribution was significantly increased in the 200 μM AFB1 group. *P = 0.03.

Mitochondria produce chemical energy in the form of ATP, which is necessary for oocyte maturation. Thus, we investigated mitochondrial distribution by Mito-Tracker staining. As shown in Fig. 5A, we found that mitochondria in the control group were distributed around the spindle, but in the AFB1 treatment group, there was a diffuse distribution or agglomeration in the cytoplasm. In addition, compared with the control group, the rate of the abnormal distribution of mitochondria in the treatment group was significantly increased (control group, 23.66 ± 4.88%, *n* = 44; AFB1 group, 61.57 ± 3.62%, *n* = 54, *P* = 0.03) (Fig. 5B). Therefore, these results indicated that AFB1 exposure led to disordered mitochondrial distribution in mouse oocytes.

## Discussion

In this study, we investigated the toxic effects of AFB1 exposure on mouse oocyte developmental capacity *via* the measurement of disturbed organelle distribution patterns. Our results showed that mouse oocyte developmental capacity was reduced after exposure to AFB1, accompanied by the abnormal arrangement of the endoplasmic reticulum, Golgi, lysosomes, and mitochondria, which might jointly represent a cause for the decreased oocyte quality in mice.

The toxicity of AFB1 was reported in different models. For mammalian oocytes, it was shown that the first polar body excretion rate of pig oocytes decreased after 50 μM AFB1 exposure (Liu et al., 2015); while melatonin could rescue the effects of 10 μM AFB1 on porcine oocyte maturation (Cheng et al., 2019). For mouse oocytes, it seemed that 50–100 μM AFB1 could effectively impact oocyte maturation (Lu et al., 2018). Our results showed that 200 μM AFB1 exposure decreased the developmental capacity of mouse oocytes by reducing the rate of polar body extrusion, indicating a decline in oocyte quality. These differences might be due to different batches of commercial products from different companies. In order to demonstrate the reason for the decline in mouse oocyte quality, we first examined the distribution of the endoplasmic reticulum (ER). The endoplasmic reticulum is closely related to the synthesis of lipids and the manufacture of membrane-bound and secretory proteins (Fagone & Jackowski, 2009). Our results showed that the distribution of the ER is disordered after exposure to AFB1. Typically, the ER should be distributed as dense rings around the chromosomes (FitzHarris, Marangos & Carroll, 2007). It has been reported that ER function may be negatively impacted by AFB1 exposure. These results have been found in primary hepatocytes of *Cyprinus carpio*, showing that the ER expands in hepatocytes when primary hepatocytes are cultured with AFB1 (0.01 μg/mL) (He et al., 2010). Moreover, co-incubation of human monocyte-derived macrophages with AFB1-generated dead cells upregulated the expression of ER stress-responsive genes in macrophages (Fishbein et al., 2020). Previous studies also showed that the ratio of abnormally distributed ER increased in mouse oocytes treated with 500 μg/L nonylphenol, accompanied by ER stress (Hu et al., 2022). When ER stress is severe or prolonged, it can lead to apoptosis (Lin et al., 2019), which may lead to a decline in oocyte developmental capacity. Our results suggested that the oocyte toxicity of AFB1 might be due to the observed abnormal distribution of ER in mice.

Proteins synthesized in the ER need to be transported to the Golgi apparatus and subsequently transferred to the cell surface by vesicular transport (Kokubun, Jin & Aoe, 2019). Therefore, we next examined the distribution of Golgi apparatus in oocytes. Previous studies reported that cigarette smoke condensate (CSC) could cause Golgi apparatus fragmentation in lung epithelial cells (Yu et al., 2016). Similarly, our results showed that the ratio of abnormal Golgi apparatus distribution was significantly elevated in the treatment group. Abnormal arrangement of Golgi apparatus is often found in oocytes exposed to harmful chemicals. Brevomycin A has been shown to disrupt Golgi apparatus, and is also known to be an inhibitor of membrane transport from the ER to the Golgi apparatus, which reduces oocyte maturation (Moreno, Schatten & Ramalho-Santos, 2002). Another study indicated that after BPA exposure, Golgi matrix protein 130 (GM130) expression was decreased in mouse oocytes, implying the destruction of Golgi function (Pan et al., 2021). Therefore, our results suggested that AFB1 exposure could affect the distribution of Golgi apparatus in mice.

Lysosomes are dynamic organelles whose functions primarily involve the degradation and processing of macromolecules, secretion, and plasma membrane repair (Oyarzun et al., 2019). They contain a variety of soluble acid hydrolases and a variety of lysosomal membrane proteins that are synthesized and transported in the ER and Golgi apparatus(Braulke & Bonifacino, 2009). Since the ER and Golgi apparatus are damaged upon AFB1 exposure, we next examined the distribution of lysosomes. Previous research has shown that AFB1 damaged lysosomes in HepG2 cells, as manifested by lysosomal alkalization (Zhang et al., 2021). Furthermore, impairment in lysosomal function has been reported to reduce the developmental capacity of porcine oocytes by disrupting the cytoskeleton and causing autophagy (Miao et al., 2019). Our results showed that the lysosomes agglutinated to form clumps following AFB1 exposure. The presence of these clumped lysosomes in oocytes represents an increase in autophagy in response to environmental stress (McGinnis, Pelech & Kinsey, 2014). Therefore, our results suggested that the clumping distribution of lysosomes may lead to their dysfunction after AFB1 exposure in mouse oocytes.

Finally, we examined mitochondria, the organelle that produces ATP for energy and interacts with other organelles through signal regulation (Chandel, 2014). Mitochondria localize around the meiotic spindle in oocytes (Malott & Luderer, 2021). The abnormal distribution of mitochondria has been found in the oocytes of diabetic mice, showing a uniform distribution. Our results showed that AFB1 exposure caused an abnormal arrangement of mitochondria in the cell. Previous studies have shown that abnormal distribution of mitochondria can impair their function. For example, cadmium exposure has been shown to reduce ATP content by disrupting mitochondrial distribution in the oocyte (Zhu et al., 2018). In addition, mitochondria are the main sites of oxidative stress and are easily attacked by both exogenous and endogenous reactive oxygen species (ROS). When ROS levels reach a high level, mitochondria are destroyed. Aflatoxin B1 exposure could increase ROS levels in primary broiler hepatocytes, reduce mitochondrial membrane potential, and ultimately induce apoptosis (Liu & Wang, 2016). Thus, these previous results and ours taken together suggest that AFB1 may affect oocyte development by causing disrupted mitochondrial distribution patterns, ultimately resulting in insufficient energy supply and exacerbating oxidative stress.

In conclusion, our study showed that AFB1 exposure to mouse oocytes disturbed the distribution of organelles, which may have induced dysfunctions of those organelles, further induing the decrease of oocyte quality.

## Supplemental Information

10.7717/peerj.13497/supp-1 Supplemental Information 1 Raw data for figures.

10.7717/peerj.13497/supp-2 Supplemental Information 2 Author checklist arrive 2.0.

## Additional Information and Declarations

### Data Availability

The following information was supplied regarding data availability: The raw measurements are available in the Supplemental File.
