# Supplementary figures and images for: Aflatoxin B1 exposure disrupts organelle distribution in mouse oocytes
Source: PeerJ. 2022 May 23;10:e13497. doi: 10.7717/peerj.13497 (PMC9135037; doi:10.7717/peerj.13497)

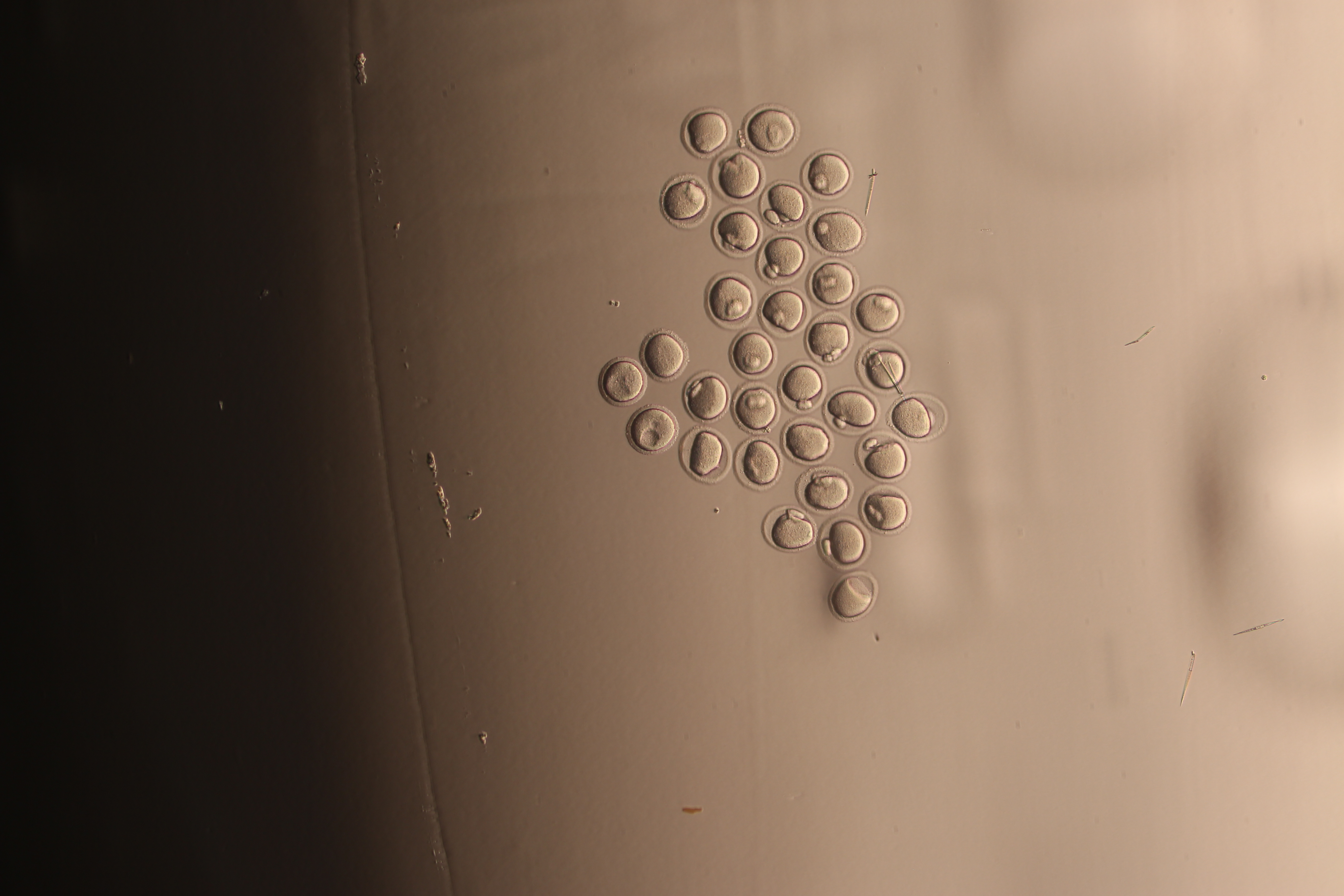

Supplement: Supplemental Information 1 [file peerj-10-13497-s001.zip › Figure1/100a╠M.JPG]

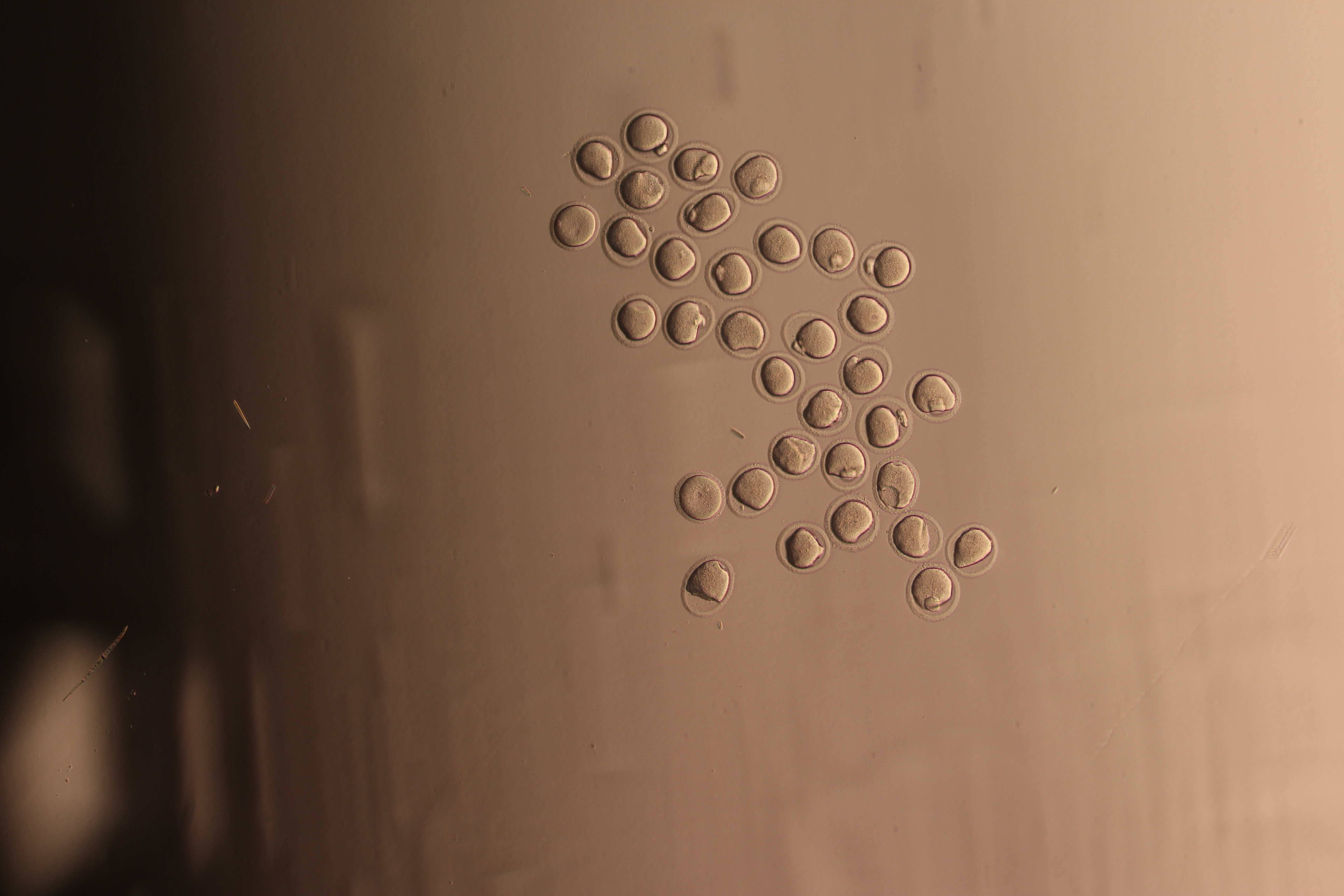

Supplement: Supplemental Information 1 [file peerj-10-13497-s001.zip › Figure1/200a╠M.JPG]

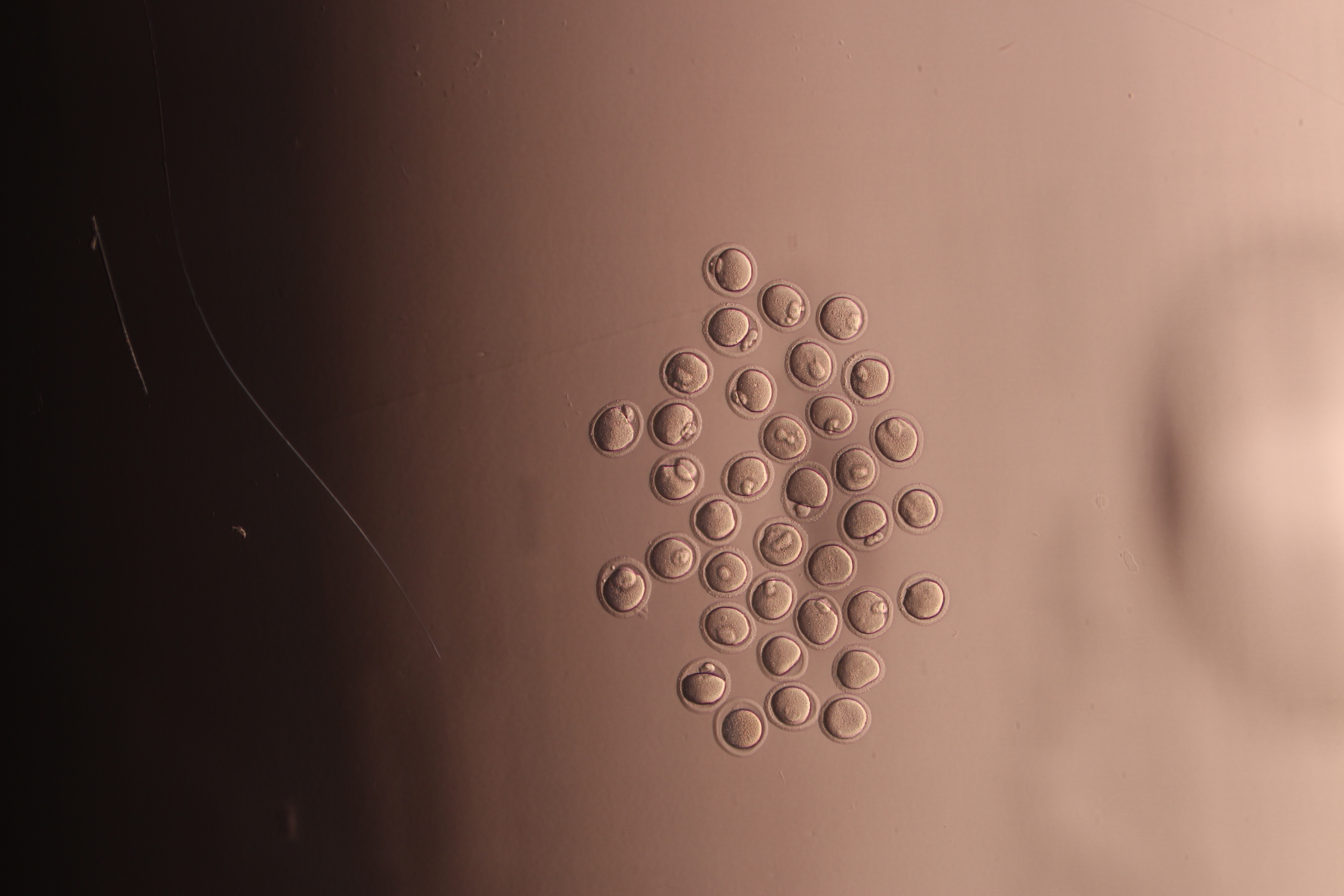

Supplement: Supplemental Information 1 [file peerj-10-13497-s001.zip › Figure1/Control.JPG]
